# Individual differences and evidence-based psychopharmacology

**DOI:** 10.1186/1741-7015-10-110

**Published:** 2012-09-27

**Authors:** RH Belmaker, Yuly Bersudsky, Galila Agam

**Affiliations:** 1 Ben Gurion University of the Negev, Beersheva, Israel

**Keywords:** Variance, clinical trials, individual differences, replicability, measurement error, variability

## Abstract

Individual differences in response to pharmacologic treatment limits the usefulness of mean data obtained from randomized controlled trials. These individual differences exist even in genetically uniform inbred mouse strains. While stratification can be of value in large studies, the individual patient history is the most effective currently available guide for personalized medicine in psychopharmacology.

## Background

**Figure 1:**
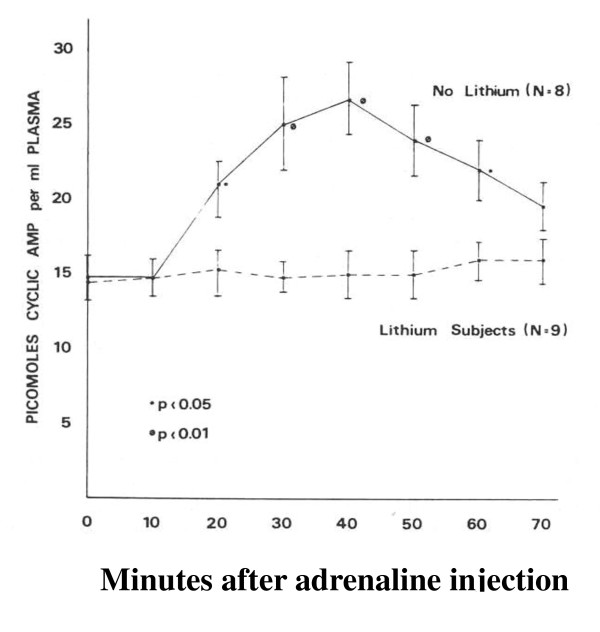
**The effect of lithium on plasma cAMP response to adrenaline administration (from **[2]**with permission)**.

**Figure 2:**
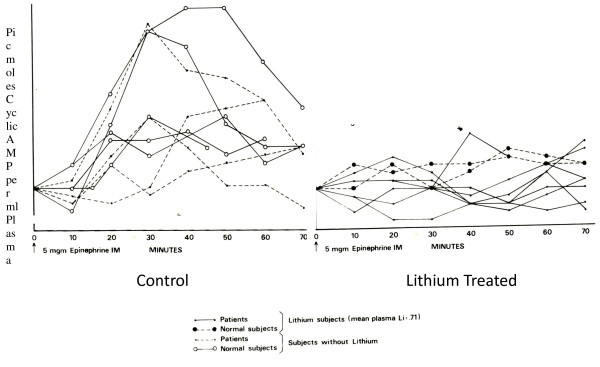
**Individual differences in the effect of lithium on plasma cAMP response to adrenaline administration (unpublished data from **[2]**)**.

The statistical nature of scientific experiment becomes clear early on in a scientist's career. While individuals without scientific background may feel that science consists of absolute facts, scientists know that all measurement involves statistical variation and that the truth involves showing a mean difference between two groups of measurements that is statistically significant, replicable, and scientifically meaningful. Medicine, however, is unique in that its focus and *raison d'être *involves the individual [1]. A physician is interested in the mean effect only to the extent that it gives him relevant information about his individual patient. If his individual patient is an outlier, his purpose as a physician is to find the appropriate intervention for that individual and the excuse that 'your results are not in accord with the average' never earned the gratitude of any patient. Thus, individual differences are the basis of medical practice rather than noise to be discarded in search of the true effect hiding behind. One of the authors became acutely aware of this after reexamining the data from his 1976 publication in Nature (see Figure 1) [2]. While the data clearly show a powerful effect, and an effect that has since been replicated, Figure 2 shows the individual differences in this effect. These individual differences might explain the current widely appreciated differences in individual response of bipolar patients to lithium treatment.

**Figure 3:**
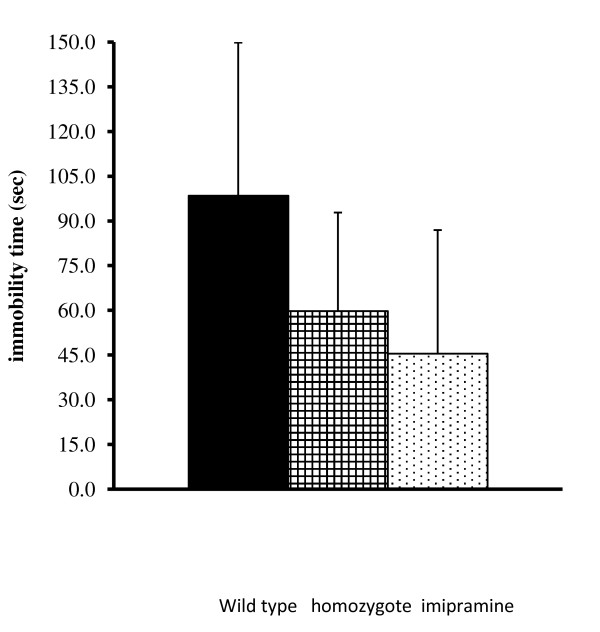
**The effect of sodium-dependent myo-inositol cotransporter homozygote knockout or imipramine on immobility after forced swim (from **[3]**with permission)**.

**Figure 4:**
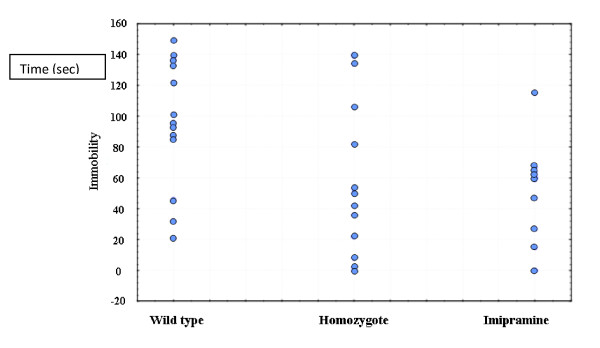
**Individual differences in the effect of sodium-dependent myo-inositol cotransporter homozygote knockout or imipramine on immobility after forced swim (unpublished data from **[3]**)**.

More recently, we have been working with knockout mice that show low levels of brain inositol due to knockout of the inositol transporter [3]: we found that these animals show a lithium-like phenotype (Figure 3). However, examination of the individual data showed the results found in Figure 4. All three groups in this study were genetically identical inbred animals handled in the same way and living in the same laboratory conditions. The individual differences cannot be ascribed to genetic effects and could not easily be ascribed to environmental effects as we commonly understand them. It is now apparent that mRNA, even in cell lines grown in culture of identical genetic origin, can vary greatly, suggesting chaotic or as yet unexplained reasons for the large individual differences [4]. The same is true of protein expression in isogenic bacterial populations [5]. These individual differences are true in much of biological and psychiatric experimentation, while they are perhaps more recognized in clinical trials and clinical experimentation.

**Figure 5:**
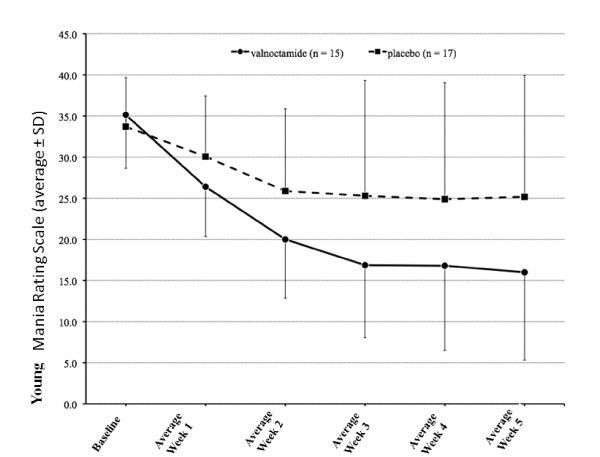
**Efficacy of valnoctamide (n = 15) versus placebo (n = 17) as an add-on to risperidone in acute mania (unpublished figure from **[6]**)**.

**Figure 6:**
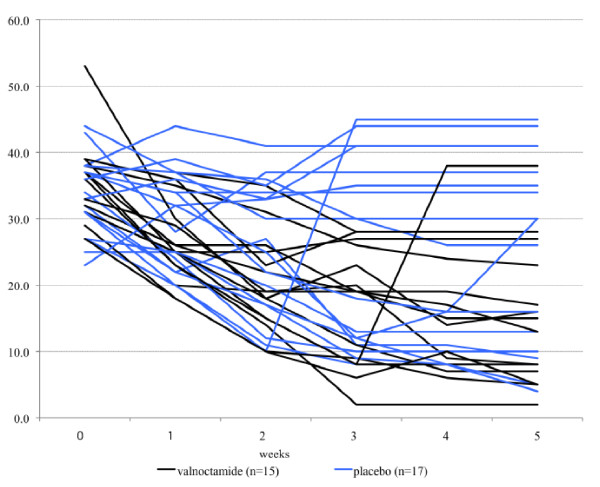
**Individual differences in efficacy of valnoctamide (n = 15) versus placebo (n = 17) as an add-on to risperidone in acute mania (unpublished data from **[6]**)**.

Often the publication style of data leads clinicians to assume that effects are more uniform than the true natural variability. We recently published the results of a small clinical trial of valnoctamide [6] as a new nonteratogenic valproate substitute (see Figure 5). While we clearly included the standard deviations and the results were statistically significant, we found that most clinicians and indeed biological scientists who have seen our paper interpret this effect as meaning that a patient treated with valnoctamide will uniformly do better than a patient treated with placebo. This is not true, as Figure 6 will immediately illustrate [6]. More patients will do well with valnoctamide than placebo but many patients on placebo will do well and many patients on valnoctamide will do poorly. The importance of making this point could be illustrated by looking at figure two in Vieta *et al*. [7]. This paper showed that treatment of bipolar depression with quetiapine, a second generation antipsychotic, led to greater mean change from baseline in depression scores than treatment with placebo. We have found that this is almost universally interpreted by clinicians into an expectation that quetiapine is an effective and powerful treatment of depression across the board for patients who meet the diagnostic criterion for bipolar depression. No standard deviations were shown to even hint at the inter-patient variability. The result is that many guidelines for the treatment of bipolar disorder list effective treatments, those treatments shown to be better than placebo, with little to guide the clinician as to make choices between those effective treatments.

## Conclusion

The truth of course is more likely that most psychiatric disorders, like most medical disorders, are highly heterogeneous [8] and that the best tool we have for dissecting that heterogeneity today is to take a careful history [9]. In the absence of a history, careful follow-up is necessary and often the best treatment for a particular patient will appear only over time. The illusion that what is needed is more head-to-head large trials without differentiating the diagnosis of bipolar depression into subtypes could lead to great expense but also to inappropriate treatment for individual patients. Medicine must return to understanding that individual differences are the essence of medicine as opposed to those sciences that are interested essentially in uncovering the grain of wheat from among all the chaff. We in medicine are interested in each bit of chaff or wheat, whoever and wherever he or she may be. Perhaps Bayes' Theorem might allow us to design more appropriate medical experiments based on this concept of individual differences. Recent papers on 'patient-centered evidence' [10] have emphasized this point as well as the need to report heterogeneity in clinical trials [11].

## Competing interests

The authors declare that they have no competing interests.

## Authors' contributions

All authors have contributed equally to this paper. All authors read and approved the final manuscript.

## Authors' information

RHB is a clinical psychopharmacologist and director of a bipolar disorder treatment unit.

YB is a clinical psychiatrist, mood disorder psychopharmacologist and biostatistician.

GA is a biochemist and neuropharmacologist and director of a biochemistry and animal behavior research laboratory.

## Pre-publication history

The pre-publication history for this paper can be accessed here:

http://www.biomedcentral.com/1741-7015/10/110/prepub
